# Proposal of an analytical method based on ELECTRE TRI to assist in establishing causal links in occupational diseases

**DOI:** 10.47626/1679-4435-2024-1338

**Published:** 2025-08-25

**Authors:** Douglas de Almeida Martins, Dalessandro Soares Vianna, Marcilene de Fátima Dianin Vianna

**Affiliations:** 1 Division of Health Expertise, Universidade Federal Fluminense (UFF), Rio das Ostras, RJ, Brazil; 2 Institute of Science and Technology, UFF, Rio das Ostras, RJ, Brazil

**Keywords:** occupational health, occupational medicine, occupational diseases, decision support techniques, surveillance of the workers’, health., saúde ocupacional, medicina do trabalho, doenças profissionais, técnicas de apoio para a decisão, vigilância em saúde do trabalhador.

## Abstract

**Introduction:**

Establishing a causal relationship between disease and work is a complex cognitive decision-making process whose effectiveness depends on the technical expertise of the physician in charge. A structured method to assist in this process could have significant practical applicability.

**Objectives:**

To develop an analytical method to support better decision-making regarding the establishment of a causal relationship between health status and occupational activities.

**Methods:**

The Design Science Research methodology was employed. In the knowledge exploration phase, the existence of analytical methods for determining a causal relationship between illness and work was investigated, along with multicriteria decision analysis techniques that could support the development of a new method. The ELECTRE TRI classification technique was selected as the basis for the analytical framework. To assess operability of the classification method, 27 simulated problem situations were analyzed. The results were found to be easy to analyze, classified as Certain, Extremely Likely, Very Likely, Likely, Plausible, Slightly Plausible, Slightly Likely, Unlikely, and Impossible. To assess efficiency, 27 case series were distributed across three questionnaires.

**Results:**

The questionnaires were completed by 56 physicians, generating a total of 504 classifications.

**Conclusions:**

By comparing the classifications assigned by the proposed method with those assigned by physicians, it was concluded that the developed method is not only innovative and practical but also an effective tool for assisting in decision-making regarding the establishment of causal relationships in occupational diseases.

## Abstract (pt)

**Introdução:**

O estabelecimento do nexo causal entre doença e trabalho é um processo cognitivo complexo de tomada de decisão, cuja eficácia depende da qualidade técnica do médico responsável. Uma proposta de método para auxiliar esse processo apresenta um grande potencial de aplicabilidade.

**Objetivos:**

Elaborar um método analítico para auxiliar a tomada de melhores decisões quanto ao estabelecimento do nexo causal entre os agravos à saúde dos trabalhadores e o exercício de suas atividades laborais.

**Métodos:**

Adotou-se a metodologia Design Science Research. Na etapa de exploração do conhecimento existente, analisou-se a existência de métodos analíticos para o estabelecimento do nexo causal e de técnicas de análise de decisão multicritério que pudessem auxiliar na elaboração do novo método. A técnica de classificação ELECTRE TRI foi selecionada para constituir o método proposto. Para avaliação da operacionalidade das classificações quanto ao nexo causal em doenças ocupacionais, foram utilizadas 27 situações-problema simuladas. Foi possível analisar facilmente os resultados, classificados nas categorias Certo, Extremamente provável, Muito provável, Provável, Plausível, Pouco plausível, Pouco provável, Improvável e Impossível. Para avaliação da eficiência, foram utilizadas 27 casuísticas, distribuídas em três questionários.

**Resultados:**

Os questionários foram respondidos por 56 médicos, totalizando 504 classificações.

**Conclusões:**

Ao comparar as classes atribuídas pelo método proposto com as atribuídas pelos médicos, concluiu-se que o método elaborado, além de inovador e prático, pode ser considerado eficiente para auxiliar na tomada de melhores decisões quanto ao estabelecimento do nexo causal em doenças ocupacionais.

## INTRODUCTION

In the daily practice of occupational physicians, determining causal relationships between workers’ health conditions and their occupational activities is a significant challenge. This decision requires a meticulous, impartial, and ethical analysis of all criteria that have effectively contributed to the deterioration of the worker’s health. Recognizing the occupational factors that contribute to workers’ illnesses requires a complex investigative process involving the assessment of several criteria and relying on the technical expertise of the physician in charge.

Establishing causal relationships between illness and work is a systemic issue in which specialist physicians should evaluate multiple criteria to determine whether to recognize this relationship. According to the Brazilian Regional Board of Medicine (*Conselho Federal de Medicina*, CFM),^1^ in addition to patient history, clinical examination (both physical and mental), medical reports, and additional tests, physicians must also consider the following factors: I - current and past clinical and occupational history, which is crucial for any diagnosis and/or investigation of causal relationships; II - investigation of the workplace; III - investigation of work organization; IV - epidemiological data; V - scientific literature; VI - occurrence of clinical or subclinical conditions in workers exposed to similar risks; VII - identification of physical, chemical, biological, mechanical, stress-related, and other hazards; VIII - testimonies and experiences from workers; and IX - knowledge and practices from other disciplines and professionals, whether or not from the health care field.

Some physicians consider only the evidence supporting the causal relationship between illness and work, while others consider only the evidence against it. There are also physicians who evaluate both supporting and opposing evidence and establish a hierarchy of relevance (weights) for all evidence.

Thus, when a medical evaluation considers only the evidence supporting the causal relationship or only the evidence against it, the outcome is a feasible decision. However, when the evaluation takes into account both supporting and opposing evidence, the result is not only feasible but also reliable, as it stems from a technical analysis that more accurately represents the true situation.

To illustrate, consider three feasible decisions in a hypothetical case of establishing a causal relationship between an adjustment disorder with anxious and depressive symptoms in a worker and their occupational activities. The first feasible decision could arise from considering only the work-related aspects that support this causality, such as productivity demands, non-negotiable targets, unattainable deadlines, lack of training, and insufficient material resources, among others. Another feasible decision could emerge from considering only the personal aspects that argue against the causal relationship, such as financial, family, and social problems, addictions, hereditary factors, sexuality issues, childhood experiences, etc. A third feasible decision would result from evaluating both professional and personal aspects. This last decision considers both the factors favoring and opposing the causal relationship. Therefore, in addition to being feasible, it would also be a reliable decision.

The first decision would likely recognize the contributing occupational factors and establish the causal relationship. For example, the worker could develop an adjustment disorder primarily due to working conditions. The second decision would likely not recognize contributing occupational factors and, consequently, would not establish the causal link. The worker might develop an adjustment disorder primarily due to personal problems, such as not residing in their birthplace where they had strong emotional ties. The third decision, however, would tend toward impartiality. The worker might develop an adjustment disorder primarily due to working conditions, even though they do not live in their birthplace, while maintaining an active social life with frequent gatherings with friends and family.

When physicians have a predetermined stance - either in favor of or against the causal relationship between illness and work - and seek only evidence that confirms their preexisting belief, they will find such evidence, regardless of its actual validity. These physicians tend to believe what they wish to believe rather than what the appropriate scientific method would indicate. This reasoning phenomenon is known in psychology as “confirmation bias” and is one of the leading causes of errors in decision-making. Nickerson^2^ defined confirmation bias as “the seeking or interpreting of evidence in ways that are partial to existing beliefs, expectations, or a hypothesis in hand.” Therefore, a new analytical method to assist in decision-making regarding causal relationships between illness and work has significant potential for applicability.

## METHODS

To develop the proposed method, the Design Science Research (DSR) methodology was employed. DSR serves as the epistemological basis for studying the “artificial.”^3,4^ According to Simon,^3^ the study of natural systems pertains to a body of knowledge about a class of objects and/or phenomena in the world, related to “how things are” (their characteristics and properties) and “how things function” (their behaviors and interactions). In contrast, the study of artificial systems, as he describes, pertains to a body of knowledge concerning “how things ought to be to attain goals.”^5^

For the operationalization of DSR, the DSR Cycle proposed by Alturki et al.^6^ was used, consisting of 15 stages: 1. Document the problem to be studied; 2. Investigate and evaluate the importance of the problem; 3. Evaluate the new solution feasibility; 4. Define research scope; 5. Define research scope within the DSR paradigm; 6. Establish type of research contribution; 7. Establish research topics; 8. Define requirements for conducting the research; 9. Define alternative solutions for the problem; 10. Explore existing knowledge; 11. Prepare for new solution design and/or evaluation; 12. Develop new solution; 13. Perform artificial evaluation of the new solution; 14. Perform naturalistic evaluation of the new solution; and 15. Communicate research findings.

In the knowledge exploration phase, the existence of analytical methods for establishing causal relationships between illness and work was investigated. A meticulous bibliometric analysis^7^ found only 1 scientific article describing a technique for establishing causal relationship in occupational diseases: the probability of causation (PC) in neoplastic diseases.^8^ Furthermore, the existence of multiple criteria decision aid (MCDA) techniques that could assist in the development of a new method was also investigated. No articles detailing an MCDA technique applicable to establishing this type of causal link were found.

**Figure 1:**
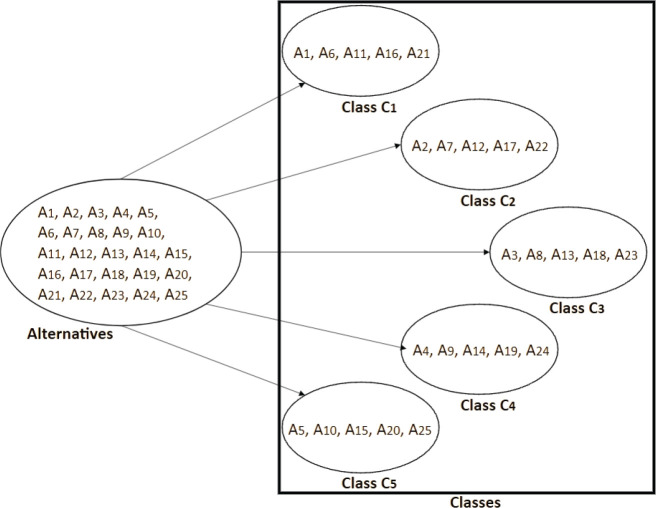
Modeling of an ordinal classification problem.

However, considering the causality scale used by Moccaldi^8^ in the setting of social security, the establishment of a causal relationship between health conditions and occupational activities can be interpreted as an ordinal classification problem, whose categories could include: Certain, Extremely Likely, Very Likely, Likely, Plausible, Slightly Plausible, Slightly Likely, Unlikely, and Impossible. Therefore, the ELECTRE TRI technique was selected as the basis for the new method. ELECTRE TRI aims to assign a set of alternatives (problem situations related to the determination of causal relationships) to specific classes based on multiple criteria and the comparison of these alternatives with the limits of each class^9^ (Figure 1).

A relevant aspect of the ELECTRE TRI class selection process is the subordination of the problem situation related to causal link determination through comparisons with the limits of each class. For better understanding, consider the following volleyball match scoreboard with 4 sets between teams A and B: A 25 x 5 B, A 20 x 25 B, A 20 x 25 B, and A 20 x 25 B. In the first set, team A performed better than team B. In the second, third, and fourth sets, team A performed worse. Therefore, team B, having won three sets, outperformed team A. The winning team is the one that wins the most sets. This is the logic of subordination; in other words, the winning (selected) class is the one that prevails in the highest number of criteria. If the logic were based on weighted averages, team A, with a higher total score, would have outperformed team B.^10^

The criteria selected for the proposed method align with the 13 requirements established by the CFM^1^: C1. medical history; C2. physical-mental evaluation; C3. reports from attending physicians; C4. additional tests; C5. clinical-occupational history; C6. Investigation of the workplace; C7. Investigation of work organization; C8. epidemiological data; C9. scientific literature; C10. knowledge from other disciplines; C11. occurrence of clinical or subclinical conditions in workers exposed to similar risks; C12. identification of occupational risks; and C13. testimony from another worker.

Each criterion has a set of characteristics. To facilitate the operationalization of the analytical method, a limited set of characteristics was established for each criterion (Chart 1).

**Chart 1:** Selected criteria for the proposed method and their characteristics

Criteria	Characteristics	
C1. Medical history	a.	Predominance of multiple work-related details (3 or more pieces of information).
	b.	Predominance of few work-related details (1 or 2 pieces of information).
	c.	No predominance of work-related or non-work-related/lifestyle details.
	d.	Predominance of few non-work-related/lifestyle details (1 or 2 pieces of information).
	e.	Predominance of multiple non-work-related/lifestyle details (3 or more pieces of information).
C2. Physical and mental evaluation	a.	Suggestive of work-related illness.
	b.	Not suggestive of work-related illness.
C3. Reports from attending physicians	a.	Predominance of multiple work-related details (3 or more pieces of information).
	b.	Predominance of few work-related details (1 or 2 pieces of information).
	c.	No predominance of work-related or non-work-related/lifestyle details.
	d.	Predominance of few non-work-related/lifestyle details (1 or 2 pieces of information).
	e.	Predominance of multiple non-work-related/lifestyle details (3 or more pieces of information).
C4. Additional tests	a.	Suggestive of work-related illness.
	b.	Not suggestive of work-related illness.
C5. Clinical-occupational history	a.	Predominance of multiple work-related details (3 or more pieces of information).
	b.	Predominance of few work-related details (1 or 2 pieces of information).
	c.	No predominance of work-related or non-work-related/lifestyle details.
	d.	Predominance of few non-work-related/lifestyle details (1 or 2 pieces of information).
	e.	Predominance of multiple non-work-related/lifestyle details (3 or more pieces of information).
C6. Workplace investigation	a.	Identification of multiple occupational factors contributing to illness (3 or more factors).
	b.	Identification of few occupational factors contributing to illness (1 or 2 factors).
	c.	No identification of occupational factors contributing to illness or not conducted.
C7. Work organization investigation	a.	Identification of multiple occupational factors contributing to illness (3 or more factors).
	b.	Identification of few occupational factors contributing to illness (1 or 2 factors).
	c.	No identification of occupational factors contributing to illness or not conducted.
C8. Epidemiological data	a.	Demonstrates strong association with work.
	b.	Demonstrates weak association with work.
	c.	Does not demonstrate association with work or non-work-related/lifestyle factors.
	d.	Demonstrates weak association with non-work-related/lifestyle factors.
	e.	Demonstrates strong with non-work-related/lifestyle factors.
C9. Scientific literature	a.	Describes strong association with work.
	b.	Describes weak association with work.
	c.	Does not describe associations with work or non-work-related/lifestyle factors.
	d.	Describes weak association with non-work-related/lifestyle factors.
	e.	Describes strong association with non-work-related/lifestyle factors.
C10. Knowledge from other disciplines	a.	Describes strong association with work.
	b.	Describes weak association with work.
	c.	Does not describe associations with work or non-work-related/lifestyle factors.
	d.	Describes weak association with non-work-related/lifestyle factors.
	e.	Describes strong association with non-work-related/lifestyle factors.
C11. Occurrence of clinical or subclinical conditions in workers exposed to similar risks	a.	Many occurrences (3 or more).
	b.	Few occurrences (1 or 2).
	c.	No occurrence.
C12. Identification of occupational risks	a.	Yes.
	b.	No.
C13. Testimonies from other workers	a.	Many testimonies highlighting a possible association between illness and work (3 or more).
	b.	Few testimonies highlighting a possible association between illness and work (1 or 2).
	c.	No testimonies highlighting a possible association between illness and work.

Thus, initially, the classes and criteria related to the ordinal classification problems concerning the causal relationship between illness and work were established. Next, the importance relationships of the ELECTRE TRI technique were defined, ie, the weights of the criteria and the limits between classes. To support neutrality in the decision-making process, these parameters were calibrated using a genetic algorithm, a search technique used in computer science and operations research to find approximate solutions in optimization problems. Developed by John Holland, this technique can be applied to optimizing decision tree learning for better performance.^11^

**Figure 2:**
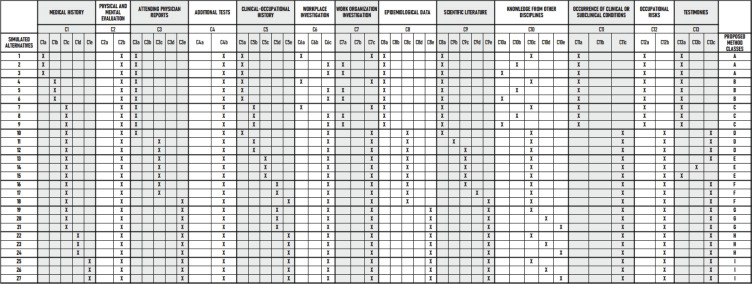
Simulated problem situations (alternatives), with the characteristics of the criteria and classes, to feed the genetic algorithm.

The genetic algorithm^12^ was initially fed with data from 27 simulated situations created by the author (Figure 2). This algorithm provided calibration data for the ELECTRE TRI parameters for application in future cases. When a satisfactory result is obtained in applying the proposed method to a case study or a real case, this problem situation and its data can be used to feed the same algorithm, contributing to the improvement of the method’s accuracy with continued use.

Thus, when faced with a clinical-occupational case requiring the establishment of a causal link between illness and work (problem situation), the physician only needs to identify the characteristics associated with the 13 criteria. To do this, the physician must answer the 13 questions and then simply apply the proposed method to obtain the classification of the problem situation regarding the causal relationship.

**Figure 3:**
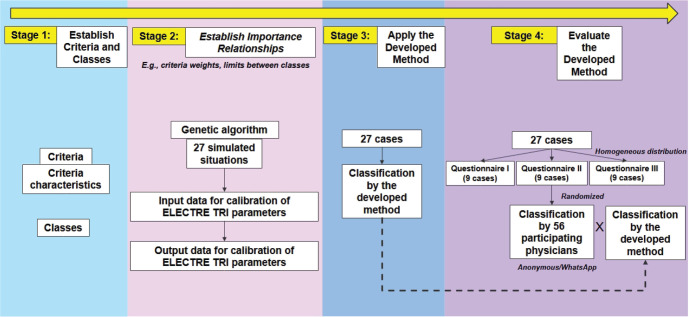
Summary of the stages of development, application, and evaluation of the proposed method.

The evaluation process of the proposed method for classification concerning causal relationships between illness and work included two stages: operationalization analysis and efficiency analysis. In the operationalization analysis, the proposed method had to be applied in a simulated environment with 27 problem situations created by the author. If it was not possible to operationalize it, the development process would need to be revised. In the efficiency analysis, the proposed method had to be applied in a real environment with 27 case studies adapted from the book *Patologia do Trabalho*.^13^ If the classifications of these 27 case studies were not predominantly congruent - that is, coinciding or corresponding in characteristics (belonging to neighboring classes) - with the classifications assigned by the occupational physicians participating in the study, the development process would also need to be revised (Figure 3). Based on this concept of congruence, the classes of the proposed method and their respective congruent classes were:

A - Certain: A, B, and C.
B - Extremely Likely: B, A, C, and D.
C - Very Likely: C, B, D, and A.
D - Likely: D, C, and B.
E - Plausible: E.
F - Slightly Plausible: F, G, and H.
G - Slightly Likely: G, H, F, and I.
H - Unlikely: H, I, G, and F.
I - Impossible: I, H, and G.

Regarding operationalization, the process was found to be easy to execute and analyze, with results classified as Certain, Extremely Likely, Very Likely, Likely, Plausible, Slightly Plausible, Slightly Likely, Unlikely, and Impossible. Regarding efficiency, it was necessary to distribute the 27 case studies homogeneously across 3 questionnaires,^12^ which were randomly answered anonymously by 56 occupational physicians after signing the Informed Consent Form,^12^ totaling 504 classifications regarding causal relationships between illness and work.

**Figure 4:**
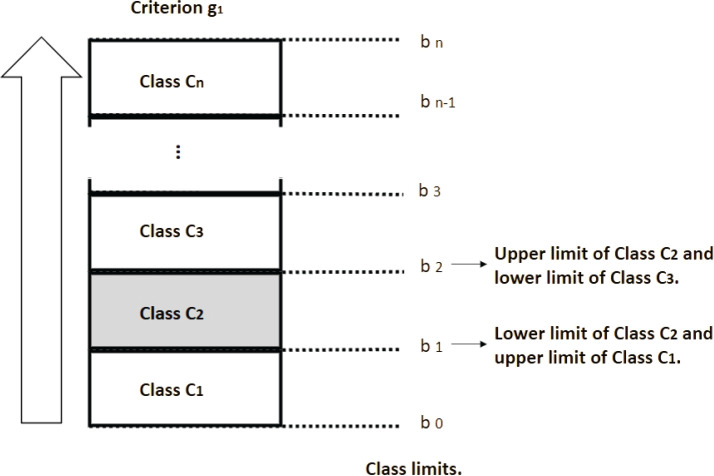
Class limits for a given criterion g_1_.

In the class selection stage using the ELECTRE TRI technique, the problem situation (alternative) was compared with the standard limits determined for the class to which it should belong (Figure 4). For this, two classification procedures were analyzed: pessimistic and optimistic. When the evaluations of a problem situation fall between the two limits of a class for each criterion, both procedures classify this problem situation within the same class; when a problem situation is incomparable for one or more limits, the pessimistic procedure classifies it in the lower class compared to the optimistic procedure.^9^ Thus, the results of the pessimistic and optimistic procedures are compared, considering the possibility of different classifications occurring. The pessimistic procedure, for example, may classify the problem situation as “Unlikely” (class H) regarding the causal link, while the optimistic procedure may classify the same problem situation as “Slightly Likely” (class G).

## RESULTS

Of the 56 participating occupational physicians, 55 (98.21%) worked in the field of occupational medicine, 45 (80.35%) had been working for more than 5 years, and 43 (76.78%) had a registered qualification as specialists.

In the pessimistic evaluation of Questionnaire I, 70.37% of responses were identical or differed by only 1 class, while 88.15% were identical or differed by up to 2 classes. If classifications with a difference of 5 or more classes were excluded from the analysis due to radical discrepancies between the classes (inconsistency), these values increased to 74.22% and 92.97%, respectively. In the optimistic evaluation of Questionnaire I, 74.07% of responses were identical or differed by only 1 class, while 88.89% were identical or differed by up to 2 classes. Excluding classifications with a difference of 5 classes or more, 76.92% of responses were identical or differed by only 1 class and 92.31% were identical or differed by up to 2 classes.

In both the pessimistic and optimistic evaluations of Questionnaire II, 69.57% of responses were identical or differed by only 1 class, while 82.13% were identical or differed by up to 2 classes. Excluding classifications with a difference of 5 or more classes, these percentages increased to 73.10% and 86.29%, respectively, in both evaluations.

In the pessimistic evaluation of Questionnaire III, 72.84% of responses were identical or differed by only 1 class, while 91.36% were identical or differed by up to 2 classes. Excluding classifications with a difference of 5 or more classes, these percentages increased to 73.29% and 91.93%, respectively. In the optimistic evaluation of Questionnaire III, 77.78% of responses were identical or differed by only 1 class, while 90.12% were identical or differed by up to 2 classes. Excluding classifications with a difference of 5 classes or more, these percentages increased to 79.25% and 91.82%, respectively.

In the pessimistic evaluation of all 3 questionnaires, 70.83% of responses were identical or differed by only 1 class, while 86.71% were identical or differed by up to 2 classes. Excluding classifications with five or more classes of difference due to inconsistencies caused by the distance between classes, these percentages increased to 73.46% and 89.92%, respectively. In the optimistic evaluation, 73.41% of responses were identical or differed by only 1 class, while 86.51% were identical or differed by up to 2 classes. After excluding classifications with five or more classes of difference, the percentages increased to 76.13% and 89.71%, respectively.

In the comparative analysis of the congruence percentage by class (Table 1), it was noted that the classes “Certain” and “Extremely Likely” had the same percentage in both evaluations (87.50% and 94.64%, respectively). In the pessimistic evaluation, the class “Very Likely” showed 100% congruence, whereas in the optimistic evaluation, this percentage was 87.83%. For the class “Likely,” congruence was 65.21% in the pessimistic evaluation and 55.26% in the optimistic evaluation.

**Table 1:** Comparative analysis of congruence percentage by class

Proposed method class	Congruent Classifications (Pessimistic Evaluation) (%)	Congruent Classifications (Optimistic Evaluation) (%)
A - Certain	87.50	87.50
B - Extremely Likely	94.64	94.64
C - Very Likely	100.00	87.83
D - Likely	65.21	55.26
E - Plausible	17.97	19.64
F - Slightly Plausible	80.00	80.00
G - Slightly Likely	76.82	76.28
H - Unlikely	89.76	91.96
I - Impossible	0.00	0.00

The class “Plausible” showed the lowest congruence percentage, considering that the developed method did not classify any cases as “Impossible.” In the pessimistic evaluation, the congruence percentage for the class “Plausible” was 17.97%, whereas in the optimistic evaluation, it was 19.64%.

It was also noted that the class “Slightly Plausible” maintained the same congruence percentage in both evaluations (80.00%). For the class “Slightly Likely,” congruence was 76.82% in the pessimistic evaluation and 76.28% in the optimistic evaluation. Meanwhile, the class “Unlikely” showed 89.76% congruence in the pessimistic evaluation and 91.96% in the optimistic evaluation.

## DISCUSSION

PC is an analytical method that estimates the risk of developing cancer due to occupational exposure to ionizing radiation.^14^ In contrast, the method proposed in this study, based on the ELECTRE TRI ordinal classification technique, is an analytical method that classifies health conditions in relation to their link to occupational activities.^15^

For PC, knowledge of population epidemiological data is mandatory.^16^ The proposed method, on the other hand, requires only the knowledge of individual data related to the characteristics of 13 criteria.

The USA, UK, Japan, and South Korea use PC for compensation claims in cases of individuals who develop illnesses due to occupational exposure to ionizing radiation.^17^ In Brazil, to date, there are no official regulations for the provision of compensation for these cases.

In countries adopting compensation systems based on PC, the recognition of occupational diseases leads to systematic developments in social security and civil and criminal justice.^17^ In Brazil, to obtain social security benefits, the establishment of a causal link between the disease and work must be carried out by an expert physician from the Brazilian Social Security National Institute.^18,19^

## CONCLUSIONS

Considering the applicability of MCDA and the difficulty in maintaining impartiality in the decision-making process for establishing a causal relationship between the health conditions of workers and their occupational activities, the analytical method proposed in this study, constituted by the ELECTRE TRI ordinal classification technique, can be considered innovative.

This method, based on answers to 13 questions related to all CFM criteria^1^ for establishing this type of link, is practical, as these responses allow classifying a case into nine classes: Certain, Extremely Likely, Very Likely, Likely, Plausible, Slightly Plausible, Slightly Likely, Unlikely, and Impossible. It is noteworthy that, in professional practice, the occupational physician responsible for the technical analysis of the causal relationship between illness and work generally cannot comply with all CFM criteria^1^ recommendations and seeks to conclude their assessment with only two classes: “There is a causal link” or “There is no causal link.”

Evaluating the congruence percentages of these classes (Table 1), the proposed method can be considered efficient in assisting the establishment of a causal relationship in any occupational disease. The development of a software that guides a technical investigation into a suspected occupational disease and alerts when there are relevant classes, such as Certain, Extremely Probable, and Very Probable, is feasible.

Finally, respecting professional autonomy, it should be noted that the proposed method does not aim to determine the causal relationship between illness and work but rather to assist in the technical investigation of suspected occupational disease. Like any technical-scientific advancement, it can and should be improved. Thus, conducting new studies on methods that can aid in establishing this type of causal link is extremely important.
